# Ethyl 1-benzyl-3-(4-methyl­phen­yl)-1*H*-pyrazole-5-carboxyl­ate

**DOI:** 10.1107/S1600536811002339

**Published:** 2011-01-22

**Authors:** Yu-Qin Li, Bao-Xiu Jia, Yu-Liang Xiao, Feng-Guang Guo

**Affiliations:** a Taishan Medical College, Tai an 271016, People’s Republic of China

## Abstract

In the title compound, C_20_H_20_N_2_O_2_, the pyrazole ring makes dihedral angles of 15.68 (4) and 83.40 (4)°, respectively, with the tolyl and benzyl rings, respectively.

## Related literature

For arelated structure, see: Ge *et al.* (2007 ▶). For applications of nitro­gen-containing heterocyclic compounds in agrochemicals and pharmaceuticals, see: Ge *et al.* (2009*a* ▶,*b* ▶, 2011 ▶). 
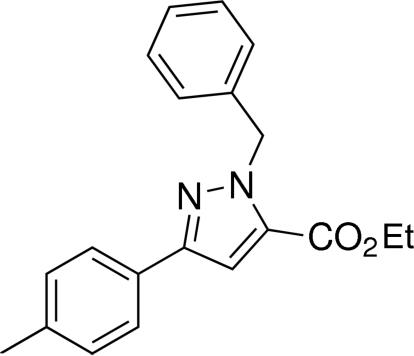


## Experimental

### 

#### Crystal data

C_20_H_20_N_2_O_2_
*M* *_r_* = 320.38
Triclinic, 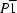

*a* = 7.666 (4) Å
*b* = 10.160 (6) Å
*c* = 11.381 (7) Å
α = 83.991 (9)°
β = 87.466 (9)°
γ = 85.47 (1)°
*V* = 878.2 (9) Å^3^
*Z* = 2
Mo *K*α radiation
μ = 0.08 mm^−1^
*T* = 298 K
0.21 × 0.16 × 0.12 mm

#### Data collection

Bruker SMART CCD area-detector diffractometer
Absorption correction: multi-scan (*SADABS*; Bruker, 1997 ▶) *T*_min_ = 0.984, *T*_max_ = 0.991
4489 measured reflections
3045 independent reflections
2182 reflections with *I* > 2σ(*I*)
*R*_int_ = 0.032

#### Refinement

*R*[*F* ^2^ > 2σ(*F* ^2^)] = 0.066
*wR*(*F* ^2^) = 0.220
*S* = 1.06
3045 reflections
217 parameters
H-atom parameters constrained
Δρ_max_ = 0.34 e Å^−3^
Δρ_min_ = −0.21 e Å^−3^

### 

Data collection: *SMART* (Bruker, 1997 ▶); cell refinement: *SAINT* (Bruker, 1997 ▶); data reduction: *SAINT*; program(s) used to solve structure: *SHELXS97* (Sheldrick, 2008 ▶); program(s) used to refine structure: *SHELXL97* (Sheldrick, 2008 ▶); molecular graphics: *SHELXTL* (Sheldrick, 2008 ▶); software used to prepare material for publication: *SHELXTL*.

## Supplementary Material

Crystal structure: contains datablocks I, global. DOI: 10.1107/S1600536811002339/jh2257sup1.cif

Structure factors: contains datablocks I. DOI: 10.1107/S1600536811002339/jh2257Isup2.hkl

Additional supplementary materials:  crystallographic information; 3D view; checkCIF report

## Figures and Tables

**Fig. 1.**
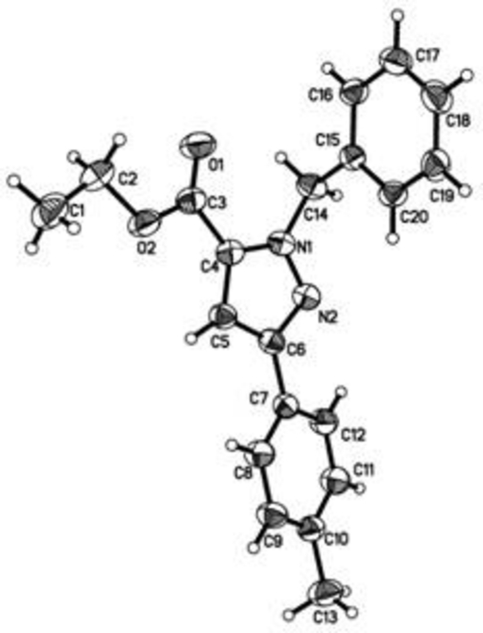
The molecular structure of (I), showing displacement ellipsoids drawn at the 50% probability level.

## supplementary crystallographic information

### Comment

Synthesis of nitrogen-containing heterocyclic compounds has been a subject of great interest due to the wide application in agrochemical and pharmaceutical fields (Ge *et al.*; 2011, 2009*a*, 2009*b*). Some pyrazole derivatives which belong to this category have been of interest for their biological activities. Considerable efforts have been devoted to the development of novel pyrazole compounds. We report here the crystal structure of the title compound, (I) (Fig. 1)

### Experimental

A mixture of ethyl 3-*p*-tolyl-1*H*-pyrazole-5-carboxylate (0.02 mol), benzyl chloride (0.0024 mol) and potassium carbonate (0.02 mol) in acetonitrile (100 ml) was heated to reflux for 10 h. The solvent was removed under reduced pressure and an product was isolated by column chromatography on silica gel (yield 82%). Crystals of (I) suitable for X-ray diffraction were obtained by slow cooling of the refluxed solution of the product in ethyl acetate at room temperature for 2 d.

### Refinement

All H atoms were placed in geometrically calculated positions and refined using a riding model with C—H = 0.97 Å (for CH_2_ groups) and 0.96 Å (for CH_3_ groups), their isotropic displacement parameters were set to 1.2 times (1.5 times for CH_3_ groups) the equivalent displacement parameter of their parent atoms.

### Crystal data

**Table:**

C_20_H_20_N_2_O_2_	*Z* = 2
*M**_r_* = 320.38	*F*(000) = 340
Triclinic, *P*1	*D*_x_ = 1.212 Mg m^−^^3^
*a* = 7.666 (4) Å	Mo *K*α radiation, λ = 0.71073 Å
*b* = 10.160 (6) Å	Cell parameters from 1778 reflections
*c* = 11.381 (7) Å	θ = 2.6–24.2°
α = 83.991 (9)°	µ = 0.08 mm^−^^1^
β = 87.466 (9)°	*T* = 298 K
γ = 85.47 (1)°	BLOCK, white
*V* = 878.2 (9) Å^3^	0.21 × 0.16 × 0.12 mm

### Data collection

**Table:**

Bruker SMART CCD area-detector diffractometer	3045 independent reflections
Radiation source: fine-focus sealed tube	2182 reflections with *I* > 2σ(*I*)
graphite	*R*_int_ = 0.032
phi and ω scans	θ_max_ = 25.1°, θ_min_ = 1.8°
Absorption correction: multi-scan (*SADABS*; Bruker, 1997)	*h* = −9→8
*T*_min_ = 0.984, *T*_max_ = 0.991	*k* = −12→9
4489 measured reflections	*l* = −13→13

### Refinement

**Table:**

Refinement on *F*^2^	Primary atom site location: structure-invariant direct methods
Least-squares matrix: full	Secondary atom site location: difference Fourier map
*R*[*F*^2^ > 2σ(*F*^2^)] = 0.066	Hydrogen site location: inferred from neighbouring sites
*wR*(*F*^2^) = 0.220	H-atom parameters constrained
*S* = 1.06	*w* = 1/[σ^2^(*F*_o_^2^) + (0.1348*P*)^2^ + 0.1525*P*] where *P* = (*F*_o_^2^ + 2*F*_c_^2^)/3
3045 reflections	(Δ/σ)_max_ < 0.001
217 parameters	Δρ_max_ = 0.34 e Å^−^^3^
0 restraints	Δρ_min_ = −0.21 e Å^−^^3^

### Special details

**Table:**

Geometry. All e.s.d.'s (except the e.s.d. in the dihedral angle between two l.s. planes) are estimated using the full covariance matrix. The cell e.s.d.'s are taken into account individually in the estimation of e.s.d.'s in distances, angles and torsion angles; correlations between e.s.d.'s in cell parameters are only used when they are defined by crystal symmetry. An approximate (isotropic) treatment of cell e.s.d.'s is used for estimating e.s.d.'s involving l.s. planes.
Refinement. Refinement of *F*^2^ against ALL reflections. The weighted *R*-factor *wR* and goodness of fit *S* are based on *F*^2^, conventional *R*-factors *R* are based on *F*, with *F* set to zero for negative *F*^2^. The threshold expression of *F*^2^ > σ(*F*^2^) is used only for calculating *R*-factors(gt) *etc*. and is not relevant to the choice of reflections for refinement. *R*-factors based on *F*^2^ are statistically about twice as large as those based on *F*, and *R*- factors based on ALL data will be even larger.

### Fractional atomic coordinates and isotropic or equivalent isotropic displacement parameters (Å^2^)

**Table:**

	*x*	*y*	*z*	*U*_iso_*/*U*_eq_	
O1	0.3059 (3)	0.5585 (2)	0.70282 (17)	0.0826 (7)	
O2	0.2483 (3)	0.6693 (2)	0.86211 (17)	0.0703 (6)	
N1	0.3539 (3)	0.3185 (2)	0.86746 (18)	0.0548 (6)	
N2	0.3537 (3)	0.2290 (2)	0.96361 (19)	0.0569 (6)	
C1	0.1960 (6)	0.9004 (4)	0.8654 (3)	0.0955 (11)	
H1A	0.1004	0.8824	0.9206	0.143*	
H1B	0.1698	0.9836	0.8191	0.143*	
H1C	0.3006	0.9049	0.9077	0.143*	
C2	0.2227 (5)	0.7932 (3)	0.7865 (3)	0.0764 (9)	
H2A	0.1213	0.7919	0.7387	0.092*	
H2B	0.3246	0.8065	0.7342	0.092*	
C3	0.2870 (3)	0.5601 (3)	0.8081 (2)	0.0597 (7)	
C4	0.3021 (3)	0.4439 (3)	0.8954 (2)	0.0528 (6)	
C5	0.2658 (3)	0.4323 (3)	1.0152 (2)	0.0551 (7)	
H5	0.2268	0.5002	1.0609	0.066*	
C6	0.2990 (3)	0.2986 (3)	1.0543 (2)	0.0517 (6)	
C7	0.2828 (3)	0.2321 (3)	1.1751 (2)	0.0523 (6)	
C8	0.2692 (4)	0.3059 (3)	1.2712 (2)	0.0625 (7)	
H8	0.2707	0.3978	1.2586	0.075*	
C9	0.2535 (4)	0.2459 (3)	1.3851 (2)	0.0669 (8)	
H9	0.2442	0.2982	1.4478	0.080*	
C10	0.2512 (4)	0.1096 (3)	1.4083 (2)	0.0616 (7)	
C11	0.2643 (4)	0.0367 (3)	1.3121 (3)	0.0717 (8)	
H11	0.2620	−0.0551	1.3247	0.086*	
C12	0.2806 (4)	0.0958 (3)	1.1980 (2)	0.0648 (8)	
H12	0.2903	0.0433	1.1355	0.078*	
C13	0.2361 (5)	0.0423 (4)	1.5327 (3)	0.0880 (10)	
H13A	0.2140	−0.0490	1.5301	0.132*	
H13B	0.1414	0.0861	1.5752	0.132*	
H13C	0.3434	0.0471	1.5719	0.132*	
C14	0.4189 (3)	0.2742 (3)	0.7545 (2)	0.0608 (7)	
H14A	0.3443	0.3163	0.6925	0.073*	
H14B	0.4110	0.1791	0.7574	0.073*	
C15	0.6048 (3)	0.3050 (2)	0.7234 (2)	0.0485 (6)	
C16	0.6535 (4)	0.3469 (3)	0.6073 (2)	0.0596 (7)	
H16	0.5692	0.3606	0.5501	0.071*	
C17	0.8246 (4)	0.3682 (3)	0.5762 (3)	0.0730 (9)	
H17	0.8563	0.3945	0.4979	0.088*	
C18	0.9483 (4)	0.3509 (4)	0.6601 (3)	0.0766 (9)	
H18	1.0637	0.3675	0.6390	0.092*	
C19	0.9039 (4)	0.3090 (3)	0.7758 (3)	0.0692 (8)	
H19	0.9892	0.2966	0.8324	0.083*	
C20	0.7326 (3)	0.2855 (3)	0.8074 (2)	0.0555 (7)	
H20	0.7025	0.2566	0.8853	0.067*	

### Atomic displacement parameters (Å^2^)

**Table:**

	*U*^11^	*U*^22^	*U*^33^	*U*^12^	*U*^13^	*U*^23^
O1	0.1089 (17)	0.0879 (17)	0.0500 (12)	−0.0083 (13)	−0.0052 (10)	−0.0005 (11)
O2	0.0972 (15)	0.0575 (12)	0.0542 (11)	−0.0067 (10)	−0.0058 (9)	0.0058 (9)
N1	0.0577 (12)	0.0589 (14)	0.0494 (12)	−0.0112 (10)	0.0018 (9)	−0.0093 (10)
N2	0.0608 (13)	0.0548 (13)	0.0561 (13)	−0.0100 (10)	0.0022 (10)	−0.0083 (10)
C1	0.131 (3)	0.061 (2)	0.091 (2)	−0.0049 (19)	−0.015 (2)	0.0112 (18)
C2	0.091 (2)	0.065 (2)	0.0695 (19)	−0.0083 (16)	−0.0136 (16)	0.0169 (16)
C3	0.0604 (16)	0.0674 (18)	0.0520 (16)	−0.0112 (13)	−0.0059 (11)	−0.0031 (13)
C4	0.0542 (14)	0.0551 (16)	0.0501 (14)	−0.0110 (11)	−0.0027 (11)	−0.0037 (12)
C5	0.0643 (16)	0.0517 (15)	0.0500 (14)	−0.0065 (12)	0.0008 (11)	−0.0082 (11)
C6	0.0545 (14)	0.0512 (15)	0.0509 (14)	−0.0089 (11)	0.0025 (11)	−0.0098 (11)
C7	0.0545 (14)	0.0491 (15)	0.0531 (14)	−0.0051 (11)	0.0026 (11)	−0.0056 (11)
C8	0.0851 (19)	0.0470 (15)	0.0561 (16)	−0.0106 (13)	0.0011 (13)	−0.0061 (12)
C9	0.089 (2)	0.0595 (18)	0.0530 (16)	−0.0082 (15)	0.0023 (14)	−0.0104 (13)
C10	0.0716 (17)	0.0582 (17)	0.0534 (15)	−0.0028 (13)	0.0007 (12)	−0.0007 (12)
C11	0.103 (2)	0.0443 (16)	0.0660 (18)	−0.0029 (14)	0.0045 (16)	−0.0023 (13)
C12	0.091 (2)	0.0462 (15)	0.0567 (16)	−0.0007 (13)	0.0029 (14)	−0.0081 (12)
C13	0.123 (3)	0.079 (2)	0.0594 (19)	−0.008 (2)	−0.0044 (18)	0.0073 (16)
C14	0.0632 (16)	0.0726 (19)	0.0512 (14)	−0.0156 (13)	−0.0016 (12)	−0.0198 (13)
C15	0.0543 (14)	0.0459 (14)	0.0472 (13)	−0.0054 (10)	−0.0033 (10)	−0.0120 (10)
C16	0.0669 (17)	0.0637 (17)	0.0481 (14)	−0.0030 (13)	−0.0076 (12)	−0.0048 (12)
C17	0.0712 (19)	0.087 (2)	0.0594 (17)	−0.0095 (16)	0.0107 (14)	−0.0026 (15)
C18	0.0563 (17)	0.092 (2)	0.081 (2)	−0.0046 (15)	0.0081 (15)	−0.0122 (18)
C19	0.0549 (16)	0.084 (2)	0.0699 (18)	0.0027 (14)	−0.0142 (13)	−0.0154 (16)
C20	0.0669 (16)	0.0551 (15)	0.0456 (13)	−0.0038 (12)	−0.0063 (11)	−0.0096 (11)

### Geometric parameters (Å, °)

**Table:**

O1—C3	1.202 (3)	C9—H9	0.9300
O2—C3	1.332 (3)	C10—C11	1.381 (4)
O2—C2	1.453 (3)	C10—C13	1.510 (4)
N1—N2	1.348 (3)	C11—C12	1.376 (4)
N1—C4	1.369 (3)	C11—H11	0.9300
N1—C14	1.461 (3)	C12—H12	0.9300
N2—C6	1.345 (3)	C13—H13A	0.9600
C1—C2	1.480 (5)	C13—H13B	0.9600
C1—H1A	0.9600	C13—H13C	0.9600
C1—H1B	0.9600	C14—C15	1.502 (3)
C1—H1C	0.9600	C14—H14A	0.9700
C2—H2A	0.9700	C14—H14B	0.9700
C2—H2B	0.9700	C15—C20	1.388 (4)
C3—C4	1.463 (4)	C15—C16	1.389 (4)
C4—C5	1.374 (3)	C16—C17	1.372 (4)
C5—C6	1.392 (4)	C16—H16	0.9300
C5—H5	0.9300	C17—C18	1.364 (4)
C6—C7	1.471 (4)	C17—H17	0.9300
C7—C12	1.384 (4)	C18—C19	1.377 (4)
C7—C8	1.386 (4)	C18—H18	0.9300
C8—C9	1.378 (4)	C19—C20	1.379 (4)
C8—H8	0.9300	C19—H19	0.9300
C9—C10	1.384 (4)	C20—H20	0.9300
			
C3—O2—C2	116.6 (2)	C11—C10—C13	121.0 (3)
N2—N1—C4	111.8 (2)	C9—C10—C13	122.1 (3)
N2—N1—C14	118.5 (2)	C12—C11—C10	122.0 (3)
C4—N1—C14	129.5 (2)	C12—C11—H11	119.0
C6—N2—N1	105.3 (2)	C10—C11—H11	119.0
C2—C1—H1A	109.5	C11—C12—C7	121.0 (3)
C2—C1—H1B	109.5	C11—C12—H12	119.5
H1A—C1—H1B	109.5	C7—C12—H12	119.5
C2—C1—H1C	109.5	C10—C13—H13A	109.5
H1A—C1—H1C	109.5	C10—C13—H13B	109.5
H1B—C1—H1C	109.5	H13A—C13—H13B	109.5
O2—C2—C1	106.8 (3)	C10—C13—H13C	109.5
O2—C2—H2A	110.4	H13A—C13—H13C	109.5
C1—C2—H2A	110.4	H13B—C13—H13C	109.5
O2—C2—H2B	110.4	N1—C14—C15	113.41 (19)
C1—C2—H2B	110.4	N1—C14—H14A	108.9
H2A—C2—H2B	108.6	C15—C14—H14A	108.9
O1—C3—O2	124.5 (3)	N1—C14—H14B	108.9
O1—C3—C4	125.5 (3)	C15—C14—H14B	108.9
O2—C3—C4	110.0 (2)	H14A—C14—H14B	107.7
N1—C4—C5	106.1 (2)	C20—C15—C16	118.7 (2)
N1—C4—C3	123.6 (2)	C20—C15—C14	121.2 (2)
C5—C4—C3	130.3 (3)	C16—C15—C14	119.9 (2)
C4—C5—C6	106.2 (2)	C17—C16—C15	120.7 (2)
C4—C5—H5	126.9	C17—C16—H16	119.7
C6—C5—H5	126.9	C15—C16—H16	119.7
N2—C6—C5	110.6 (2)	C18—C17—C16	119.9 (3)
N2—C6—C7	120.6 (2)	C18—C17—H17	120.0
C5—C6—C7	128.8 (2)	C16—C17—H17	120.0
C12—C7—C8	117.3 (2)	C17—C18—C19	120.6 (3)
C12—C7—C6	122.4 (2)	C17—C18—H18	119.7
C8—C7—C6	120.2 (2)	C19—C18—H18	119.7
C9—C8—C7	121.3 (3)	C18—C19—C20	119.7 (3)
C9—C8—H8	119.4	C18—C19—H19	120.1
C7—C8—H8	119.4	C20—C19—H19	120.1
C8—C9—C10	121.5 (3)	C19—C20—C15	120.3 (2)
C8—C9—H9	119.3	C19—C20—H20	119.9
C10—C9—H9	119.3	C15—C20—H20	119.9
C11—C10—C9	116.9 (3)		
			
C4—N1—N2—C6	0.6 (3)	C12—C7—C8—C9	−0.2 (4)
C14—N1—N2—C6	176.1 (2)	C6—C7—C8—C9	179.8 (2)
C3—O2—C2—C1	−175.4 (3)	C7—C8—C9—C10	0.2 (5)
C2—O2—C3—O1	1.8 (4)	C8—C9—C10—C11	−0.4 (5)
C2—O2—C3—C4	−177.9 (2)	C8—C9—C10—C13	179.3 (3)
N2—N1—C4—C5	−0.6 (3)	C9—C10—C11—C12	0.6 (5)
C14—N1—C4—C5	−175.4 (2)	C13—C10—C11—C12	−179.1 (3)
N2—N1—C4—C3	−178.8 (2)	C10—C11—C12—C7	−0.7 (5)
C14—N1—C4—C3	6.4 (4)	C8—C7—C12—C11	0.4 (4)
O1—C3—C4—N1	5.4 (4)	C6—C7—C12—C11	−179.6 (3)
O2—C3—C4—N1	−174.9 (2)	N2—N1—C14—C15	−99.0 (3)
O1—C3—C4—C5	−172.3 (3)	C4—N1—C14—C15	75.5 (3)
O2—C3—C4—C5	7.4 (4)	N1—C14—C15—C20	42.9 (4)
N1—C4—C5—C6	0.4 (3)	N1—C14—C15—C16	−140.7 (3)
C3—C4—C5—C6	178.4 (2)	C20—C15—C16—C17	0.2 (4)
N1—N2—C6—C5	−0.4 (3)	C14—C15—C16—C17	−176.3 (3)
N1—N2—C6—C7	−179.9 (2)	C15—C16—C17—C18	−1.3 (5)
C4—C5—C6—N2	0.0 (3)	C16—C17—C18—C19	1.5 (5)
C4—C5—C6—C7	179.5 (2)	C17—C18—C19—C20	−0.6 (5)
N2—C6—C7—C12	−16.0 (4)	C18—C19—C20—C15	−0.5 (4)
C5—C6—C7—C12	164.6 (3)	C16—C15—C20—C19	0.7 (4)
N2—C6—C7—C8	164.0 (2)	C14—C15—C20—C19	177.2 (2)
C5—C6—C7—C8	−15.4 (4)		
